# Dollo-CDP: a polynomial-time algorithm for the clade-constrained large Dollo parsimony problem

**DOI:** 10.1186/s13015-023-00249-9

**Published:** 2024-01-08

**Authors:** Junyan Dai, Tobias Rubel, Yunheng Han, Erin K. Molloy

**Affiliations:** 1 Department of Computer Science, University of Maryland, College Park, MD USA; 2 University of Maryland Institute for Advanced Computer Studies, College Park, MD USA

**Keywords:** Phylogenetics, Parsimony, Dollo, Retrotransposons

## Abstract

The last decade of phylogenetics has seen the development of many methods that leverage constraints plus dynamic programming. The goal of this algorithmic technique is to produce a phylogeny that is optimal with respect to some objective function and that lies within a constrained version of tree space. The popular species tree estimation method ASTRAL, for example, returns a tree that (1) maximizes the quartet score computed with respect to the input gene trees and that (2) draws its branches (bipartitions) from the input constraint set. This technique has yet to be used for parsimony problems where the input are binary characters, sometimes with missing values. Here, we introduce the clade-constrained character parsimony problem and present an algorithm that solves this problem for the Dollo criterion score in $O(|\Sigma |^{3.726}(n+k) + |\Sigma |^{1.726}nk)$ time, where *n* is the number of leaves, *k* is the number of characters, and $\Sigma$ is the set of clades used as constraints. Dollo parsimony, which requires traits/mutations to be gained at most once but allows them to be lost any number of times, is widely used for tumor phylogenetics as well as species phylogenetics, for example analyses of low-homoplasy retroelement insertions across the vertebrate tree of life. This motivated us to implement our algorithm in a software package, called Dollo-CDP, and evaluate its utility for analyzing retroelement insertion presence / absence patterns for bats, birds, toothed whales as well as simulated data. Our results show that Dollo-CDP can improve upon heuristic search from a single starting tree, often recovering a better scoring tree. Moreover, Dollo-CDP scales to data sets with much larger numbers of taxa than branch-and-bound while still having an optimality guarantee, albeit a more restricted one. Lastly, we show that our algorithm for Dollo parsimony can easily be adapted to Camin-Sokal parsimony but not Fitch parsimony.

**Supplementary Information:**

The online version contains supplementary material available at 10.1186/s13015-023-00249-9.

## Introduction

The last decade of phylogenetics has seen the development of many methods that leverage constraints plus dynamic programming (CDP). The goal of CDP is to produce a phylogeny that is optimal with respect to some objective function and that lies within a constrained version of tree space. To our knowledge, the first method based on CDP was introduced in 2000 by Hallet and Lagergren [1] for gene tree parsimony, which seeks a species tree that minimizes the number of events (e.g., duplications and losses) needed to explain the input gene trees (also see the related results presented at WABI 2017 [2, 3]). Since its introduction, CDP has been leveraged for a variety of optimization problems, including minimizing deep coalescence [4], maximizing quartet support [5, 6] (see [7] for extensions to multi-copy genes), and maximizing bipartition support [8] (see [9] for extensions to multi-copy genes). All of these optimization problems take gene trees as input and seek a species tree that minimizes the dissimilarity between it and the input gene trees or alternatively maximizes the similarity (note that species trees depict the evolutionary history of species, whereas gene trees depict the evolutionary history of recombination-free, orthologous genomic regions).

That CDP is so widely utilized in phylogenetics is likely due to it being possible to build effective constraints in practice. The constraints are effective if (nearly) optimal solutions lie within the constrained search space and this space is small enough to enable efficient running times. As an example, the species tree estimation method ASTRAL solves the *bipartition-constrained maximum quartet support supertree problem* [6]. The first version of ASTRAL [6] formed the constrained solution space from the set of all bipartitions (i.e., branches) found in the input gene trees. The newer versions of ASTRAL [10, 11] allow extra bipartitions to be included as constraints with the goal of improving accuracy. FASTRAL [12], on the other hand, aggressively limits the number of bipartitions added with the goal of improving runtime. Overall, the popularity of the ASTRAL family of methods is likely due to their speed and accuracy on practical inputs.

Given the success of CDP thus far, we explore the use of this technique for traditional parsimony problems where the input are binary characters, sometimes with missing values. The remainder of this paper is organized as follows. After providing notation and preliminaries, we introduce the *clade-constrained character parsimony problem* and present an algorithm that solves this problem in polynomial time for the Dollo criterion score. We then show how our algorithm can be adapted to Camin-Sokal parsimony but not Fitch parsimony. Dollo parsimony, in particular, is widely used for tumor phylogenetics [13–16] as well as species phylogenetics, for example analyses of the presence or absence of low-homoplasy retroelement insertions across the vertebrate tree of life. Prior studies have leveraged Dollo parsimony to analyze higher-level clades of birds (e.g., *Palaeognathe* [17]) and mammals (e.g., *Laurasiatheria* [18, 19]), in addition to clades at the family and genus levels (e.g., rorquals [20], mouse-eared bats [21, 22], and primates [23]).

This motivated us to implement our algorithm for the Dollo criterion score in Dollo-CDP, an open-source software package available on Github. We evaluate Dollo-CDP on real and synthetic data sets of retroelement insertion presence / absence in comparison to branch-and-bound and heuristic search. Our results reveal that Dollo-CDP can improve upon heuristic search from a single starting tree, often recovering a better scoring tree. Moreover, Dollo-CDP scales to data sets with much larger numbers of taxa than branch-and-bound while still having an optimality guarantee, albeit a more restricted one. We conclude with a discussion of limitations and opportunities for future research.

## Background

Before introducing the clade-constrained Dollo parsimony problem, we review some preliminaries on phylogenetic trees, characters, and parsimony approaches.

### Phylogenetic trees

A *phylogenetic tree* *T* is an acyclic graph whose leaves (i.e., vertices with degree one) are bijectively labeled by a set *S* of species (note that in the context of tumor phylogenetics the leaves may be labeled by cells in a tumor). For convenience and simplicity of notation, we treat leaves and species as being interchangeable. We use *L*(*T*), *V*(*T*), and *E*(*T*) to denote the leaf set, vertex set, and edge set of *T*, respectively.

Phylogenetic trees can be either *unrooted* or *rooted*; for the former the graph is undirected and for the latter the graph is directed, with edges orientated away from the root (a special vertex with in-degree zero). For rooted trees, we say that vertex *u* is an ancestor of *v* (or that *v* is a descendant of *u*) if *u* is on a directed path from the root to *v*. The *lowest common ancestor (LCA)* for a set *V* of vertices is the vertex that is the ancestor of all vertices in *V* that is farthest away from the root.

Unless otherwise noted, we will assume that all trees are *binary*. An unrooted tree is binary if all non-leaf vertices (called internal vertices) have degree three, and a rooted tree is binary if all non-leaf vertices have out-degree two (all non-root vertices have in-degree one). For rooted trees, we use *v*.*parent* to indicate the parent of vertex *v*; similarly, we use *v*.*left* and *v*.*right* to denote the left and right children of vertex *v*, respectively. Sometimes it will be useful to restrict a tree *T* to a subset $X \subseteq S$ of leaves, meaning that each leaf in $S {\setminus } X$ is deleted from *T* and then all vertices with degree two are suppressed. There are three additional concepts for phylogenetic trees that will prove useful later.

#### Definition 1

(Bipartition) Each edge *e* in an unrooted phylogenetic tree *T* induces a *bipartition*, which splits the leaf set of *T* into two disjoint subsets whose union is the complete leaf set *S*. The bipartition $Bip(e) = X|Y$ is formed by deleting edge *e* but not its endpoints from *T* and assigning the leaves in one of the resulting subtrees to *X* and the leaves in the other to *Y*.

#### Definition 2

(Clade) Each vertex *v* in a rooted phylogenetic tree *T* induces a *clade*, denoted *Clade*(*v*), which is simply the set of leaves that are descendants of *v*. A clade is *trivial* if it contains only a single element (as it must be associated with a leaf vertex) or if it contains all leaves (as it must be associated with the root vertex).

#### Definition 3

(Subtree bipartition [24]) Each internal vertex *v* in a rooted binary phylogenetic tree *T* induces a *subtree bipartition*, which partitions the leaf set of the subtree into two disjoint subsets whose union is *Clade*(*v*). A subtree bipartition $SubBip(v) = X|Y$ is formed by setting *X* to be the leaves that are descendants of *v*.*left* and *Y* to be the set of leaves that are descendants of *v*.*right* (or vice versa).

It is well established that an unrooted phylogenetic tree *t* is uniquely defined by its bipartition set $Bip(t) = \{Bip(e): e \in E(t) \}$ and that a rooted phylogenetic tree *T* is uniquely defined by its clade set $Clade(T) = \{Clade(v): v \in V(T) \}$ (see Chapters 2 and 3 in [25]). Similarly, we define the subtree bipartition set of *T* as $SubBip(T) = \{SubBip(v): v \in V(T) \}$. Note that $X|Y \in SubBip(T)$ if and only if $\{X, Y, X \cup Y\} \subseteq Clade(T)$; thus, we can go back and forth between clades and subtree bipartitions.

### Characters and parsimony

A *character* *c* on species set *S* is a function mapping species in *S* to a state set, which is $\{\texttt {0}, \texttt {1}\}$ for binary characters. For biological data, the 0 and 1 might refer to some feature of the genomic data with all species assigned the same state having the same feature. If $\texttt {0}$ indicates the ancestral state and $\texttt {1}$ indicates the derived (i.e., mutated) state, we say the characters are *ordered*; otherwise, we say they are *unordered*. We later describe how biological data that are encoded as ordered binary characters. These character matrices also include a third state ? to indicate the state assignment is ambiguous or missing; in other words, it could not be reliably called as $\texttt {0}$ or $\texttt {1}$.

For now, we assume that we are given a set ${\mathcal {C}}$ of binary characters with no missing values, and our goal is to find a phylogenetic tree *T* that best explains our data. To explain how character *c* evolves on a tree *T* on the same species set as *c*, we must assign states to the internal vertices of *T*. The quality of our explanation is determined by the number of substitutions, where a substitution is implied by any edge $e = (u,v) \in E(T)$ such that $c[u] \ne c[v]$ (assuming neither *c*[*u*] nor *c*[*v*] are the ambiguous state $\texttt {?}$). This brings us to the small and large parsimony problems.

#### Definition 4

(Small Fitch Parsimony Problem) Given an unrooted binary tree *T* and an unordered binary character *c*, both on species set *S*, the *Fitch parsimony score*, denoted *Fitch*(*T*, *c*), is the minimum number of substitutions needed to explain the evolution of *c* on *T*. A *Fitch-labeling* for (*T*, *c*) is a function ${\hat{c}}$ mapping vertices in *V*(*T*) to states in $\{0, 1\}$ so that ${\hat{c}}[l] = c[l]$ for all $l \in L(T)$ and the number of substitutions equals *Fitch*(*T*, *c*).

#### Problem 1

(Large Fitch Parsimony Problem) The *large Fitch parsimony problem* takes as input a set ${\mathcal {C}}$ of unordered binary characters, each on species set *S*; the output is an unrooted binary tree *T* on *S* that minimizes $\sum _{c \in {\mathcal {C}}} Fitch(T,c)$.

Although the small Fitch parsimony problem can be solved in polynomial time [26], the large Fitch parsimony problem is NP-hard [27]. We now consider ordered characters and rooted phylogenetic trees, which enables us to distinguish between the $\texttt {0} \rightarrow \texttt {1}$ substitution (indicating a mutation is *gained*) and the $\texttt {1} \rightarrow \texttt {0}$ substitution (indicating a mutation is *lost*).

#### Definition 5

(Dollo and Camin-Sokal Parsimony Score) Given a rooted binary tree *T* and an ordered binary character *c*, both on species set *S*, the *Dollo parsimony score*, denoted *Dollo*(*T*, *c*), is the minimum number of losses needed to explain the evolution of *c* on *T* when at most one gain is allowed (note that sometimes the gains are also counted as part of the score). The *Camin-Sokal parsimony score*, denoted *CamSok*(*T*, *c*), is the minimum number of gains needed to explain the evolution of *c* on *T* when losses are prohibited.

Just as the Fitch parsimony score was used to define the Fitch-labeling and the large Fitch parsimony problem, we can define similar concepts for the Dollo and Camin-Sokal parsimony scores. The following result about Dollo-labelings is the basis of a linear-time algorithm presented by Bouckaert et al. [28] for computing *Dollo*(*T*, *c*).

#### Theorem 1

(Theorem 1 in [28]) Let *T* be a rooted, binary tree and let *c* be an ordered, binary character with no missing values, both on the same species set. A Dollo-labeling for (*T*, *c*) must assign state 1 to every internal vertex on a path from the LCA of all leaves assigned state 1 (including the LCA) to any leaf assigned state 1 and assign state 0 to all remaining vertices.

**Fig. 1:**
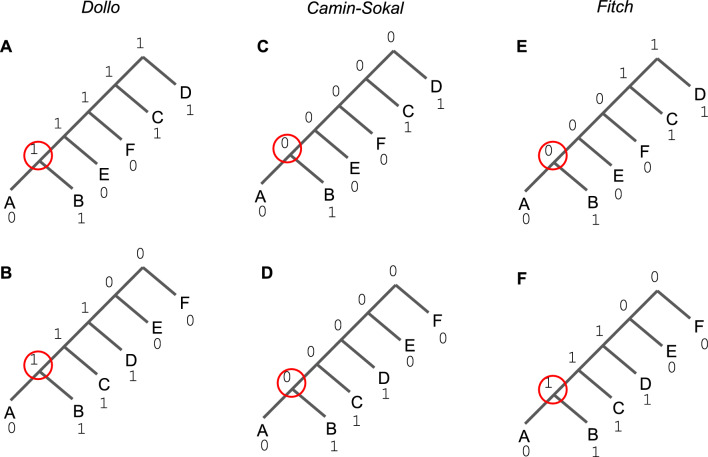
Let *v* be the internal vertex associated with the subtree bipartition *X*|*Y*, where $X = \{A\}$ and $Y = \{B\}$ (note that these vertices are circled in the trees above). Subfigures **A** and **B** show two different trees on the same species set with Dollo-labelings for the same character. The state assignment at *v* only requires us to know the subtree bipartition associated with *v* (Corollary 1). Vertex *v* is assigned state 1 because there is a leaf in *Y* assigned state 1 and a leaf in $S \setminus X \cup Y$ assigned state 1. Subfigures **C** and **D** show two different trees with Camin-Sokal-labelings for the same character. The state assignment at *v* only requires us to know the clade associated with *v* (Corollary 3). Vertex *v* is assigned state 0 because there is a leaf in clade $X \cup Y$ assigned state 0. Lastly, subfigures **E** and **F** show two different trees with Fitch-labelings for the same character. In subfigure **E**, the assignment of state 0 or 1 to *v* results in a score of two or three, respectively (so 0 is better). In subfigure **F**, the assignment of state 0 or 1 to *v* results in a score of three or two, respectively (so 1 is better). Thus, for the Fitch criterion score, the state assignment at *v* depends on more than the bipartition induced by the edge incident to *v*

It follows that a Dollo-labeling for (*T*, *c*) is unique and always exists, provided the root is allowed to be assigned state 1. In this case, the gain must have occurred above the root so no gains are allowed on *T* (see Fig. 1A for an example).

Lastly, the large Dollo and Camin-Sokal parsimony problems are NP-hard [29]. The existing methods for these problems, including those implemented in PAUP* [30] and Phylip [31], are based on either heuristic searches of tree space (which have no optimality guarantees) or branch-and-bound (which is guaranteed to find an optimal solution but is time consuming for large, complex data sets). We provide an overview of these approaches in presenting our experimental study, referring the interested reader to [32, 33] for more information.

## The clade-constrained large Dollo parsimony problem and a polynomial-time algorithm

We now introduce the *clade-constrained large Dollo parsimony* (CC-LDP) problem.

### Problem 2

(Clade-constrained large Dollo parsimony problem) The CC-LDP problem is defined by the following input and output.

**Input:** A set *S* of species, a set ${\mathcal {C}}$ of ordered binary characters (each on species set *S*), and a set $\Sigma$ of clades (subsets of *S*)

**Output:** A rooted binary tree on species set *S* such that $\sum _{c \in {\mathcal {C}}} Dollo(T,c)$ is minimized and $Clade(T) \subseteq \Sigma$, if such a tree exists

The CC-LDP problem has a solution provided there exists at least one binary tree *T* on *S* with $Clade(T) \subseteq \Sigma$. In our experimental study, we present approaches for constructing $\Sigma$ from ${\mathcal {C}}$ in such a way that a solution to CC-LDP always exists. The basis of our polynomial-time algorithm for CC-LDP is that for an arbitrary tree *T*, the Dollo-labeling for every character in ${\mathcal {C}}$ at an internal vertex *v* of *T* is fully constrained by the subtree bipartition induced by *v*. Thus, the clade set $\Sigma$ not only constrains the search space in terms of the allowed tree topologies but also the allowed state assignments at internal vertices. This decouples the processing of individual characters for the NP-hard *large* Dollo parsimony problem, similar to the *small* Dollo parsimony problem, which, as previously mentioned, can be solved in polynomial time (e.g. via the Sankoff-Rousseau [34] algorithm). We begin by giving a corollary of Theorem 1 in [28].

### Corollary 1

Let *c* be an ordered binary character on species set *S* (with no missing values), let *T* be a rooted binary tree on *S*, and let ${\hat{c}}$ be the unique Dollo-labeling for (*T*, *c*). Then for any internal vertex $v \in V(T)$, ${\hat{c}}[v]$ can be determined just by having knowledge of its subtree bipartition *SubBip*(*v*); no other information about *T* is needed.

### Proof

Consider an arbitrary internal vertex $v \in V(T)$ that induces subtree bipartition *X*|*Y*. Without loss of generality, let *X* contain the leaves that are descendants of *v*.*left*, let *Y* contain the leaves that are descendants of *v*.*right*, and let *Z* contain all other leaves so *X*|*Y*|*Z* is a partition of *S*. When *c* has no missing values, by Theorem 1, there exists a unique Dollo-labeling ${\hat{c}}$ for (*T*, *c*), where ${\hat{c}}[v] = \texttt {1}$ if at least one of the following two cases holds (otherwise ${\hat{c}}[v] = \texttt {0}$).

- **Case A:** Vertex *v* is the LCA of two leaves assigned state 1.
- **Case B:** Vertex *v* is on the path from the LCA of two leaves assigned state 1 to one of those two leaves.

Looking at subtree bipartition $SubBip(v) = X|Y$, case A holds if condition 1 below is true, and case B holds if at least one of conditions 2 and 3 below is true.

- **Condition 1:** There exists a leaf $x \in X$ and a leaf $y \in Y$ such that $c[x] = c[y] = \texttt {1}$.
- **Condition 2:** There exists a leaf $x \in X$ and a leaf $z \in Z$ such that $c[x] = c[z] = \texttt {1}$.
- **Condition 3:** There exists a leaf $y \in Y$ and a leaf $z \in Z$ such that $c[y] = c[z] = \texttt {1}$.

Therefore, ${\hat{c}}[v] = \texttt {1}$ if at least one of conditions 1–3 is true; otherwise ${\hat{c}}[v] = \texttt {0}$. Conditions 1–3 can be evaluated so long as we know the subtree bipartition induced by *v* (given the character *c* and its complete leaf set *S*). $\square$

### Theorem 2

Let *c* be an ordered binary character on species set *S*, and let *T* be a rooted binary tree on *S*. If *c* has missing values, the Dollo-labeling for (*T*, *c*) may not be unique. However, we can define a unique Dollo-labeling ${\hat{c}}_*$ for (*T*, *c*) with the property that ${\hat{c}}_*[v]$ can be determined for any internal vertex $v \in V(T)$ just by having knowledge of its subtree bipartition *SubBip*(*v*); no other knowledge of *T* is needed.

### Proof

If *c* contains missing values or ambiguous states, we define *R* to be the subset of leaves assigned non-ambiguous states, letting $T|_R$ and $c|_R$ denote the restriction of *T* and *c* to *R*, respectively. We claim that *Dollo*(*T*, *c*) must equal $Dollo(T|_R, c|_R)$. To prove this claim, we first show how to construct a labeling ${\hat{c}}$ for (*T*, *c*) that yields score $Dollo(T|_R, c|_R)$ given a Dollo-labeling ${\hat{c}}|_R$ for $(T|_R,c|_R)$. We then show that ${\hat{c}}$ is a Dollo-labeling for (*T*, *c*).

**Fig. 2:**
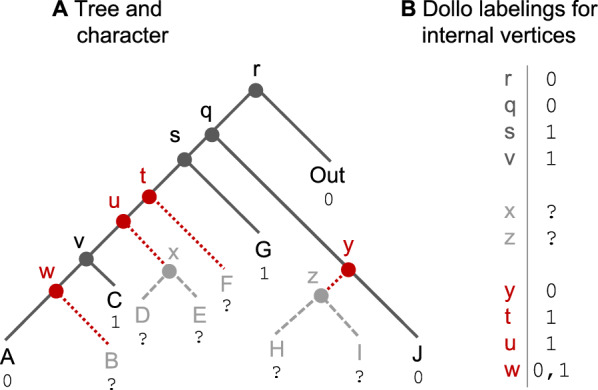
When there are ambiguous states, we can restrict a character *c* and tree *T* to the subset of labels assigned non-ambiguous states (i.e., $R = \{A, C, G, J, Out\}$) and then compute the Dollo score in the usual fashion (see proof of Theorem 2). To create the restricted tree $T|_R$, we first identify all edges incident to maximally-sized subtrees with all leaves assigned the ambiguous state (shown in red shorter dashes). After deleting these edges, we have a tree $T'$ on *R* (shown with solid lines) and a collection *P* of subtrees (shown in grey, with edges as longer dashes) whose leaves are all assigned the ambiguous state. We then suppress vertices with out-degree 1 (shown in red) in $T'$ to get $T|_R$. Lastly, we apply conditions 1–3 to find the Dollo-labeling for the internal vertices of $T|_R$; this gives us one loss on edge $v \mapsto A$ (and also one gain on edge $q \mapsto s$). This procedure for constructing $T|_R$ classifies vertices in *T* into three groups. The vertices in **Group 1** (*r*, *q*, *s*, *v*) are assigned the same labels as in $T|_R$. The vertices in **Group 2** (*x*, *z*) are assigned the ambiguous state. The vertices in **Group 3** (*t*, *u*, *w*, *y*) need to be assigned states so as not to increase the Dollo score. In this example, there are two possible ways to assign a state to vertex *w*; the approach described in the proof of Theorem 2 assigns state 0 to *w*

To build ${\hat{c}}$, we classify the vertices of *T* into three groups based on the formation of $T|_R$ described next (see Fig. 2 for an example).

1. First, we identify every edge incident to the root of a maximally-sized subtree of *T* whose leaves are all assigned state ?. That is, we identify every edge $a \mapsto b \in E(T)$ such that all leaves in *Clade*(*b*) are assigned state ? *and* at least one leaf in *Clade*(*a*) is assigned a non-ambiguous state. Let ${\mathcal {E}}$ denote the set of edges with this property.
2. Second, we delete each edge in ${\mathcal {E}}$ but not its endpoints from *T*. This produces a tree $T'$ with no leaves assigned state $\texttt {?}$ plus a collection ${\mathcal {P}}$ of subtrees of *T* whose leaves are all assigned state $\texttt {?}$.
3. Lastly, we form $T|_R$ by suppressing all vertices in $T'$ with out-degree one.

We now use the above procedure to classify the vertices of *T* into three groups by which we can build a labeling ${\hat{c}}$ for (*T*, *c*) that yields score $Dollo(T|_R, c|_R)$ as follows.

- **Group 1** contains every vertex $v \in V(T)$ that maps to a vertex $w \in V(T|_R)$. For each vertex *v* in this group, we assign ${\hat{c}}[v] = {\hat{c}}|_R[w]$. The idea is to propagate the state assignments for $T|_R$ back to *T*. Now we need to assign states to the remaining vertices in *T* without changing the Dollo score.
- **Group 2** contains every vertex $v \in V(T)$ that maps to a vertex $w \in \cup _{t \in {\mathcal {P}}} V(t)$, meaning that *v* is in a maximally-sized subtree of *T* whose leaves are all assigned state ?. For each vertex *v* in this group, we set ${\hat{c}}[v] = \texttt {?}$. This state assignment does not change the Dollo score because substitutions are not counted on edges with at least one of their two endpoints assigned state ?.
- **Group 3** contains every vertex $v \in V(T)$ that does not map to any vertex in $V(T|_R) \cup \cup _{t \in {\mathcal {P}}} V(t)$, meaning that the corresponding vertex must have been suppressed in step 3. Thus, *v* maps to an edge $e \in E(T|_R)$, and exactly one of *v*’s children is the root of a maximally-sized subtree of *T* whose leaves are all assigned state ?. A state assignment that does not change the Dollo score can be achieved by assigning every vertex *v* in this group that maps back to the same edge $e = s \mapsto t \in E(T|_R)$ the same state: either the state assigned to *s* or the state assigned to *t*.

Importantly, there can be multiple ways to assign states to vertices in group 3; thus, a unique labeling for (*T*, *c*) that yields score $Dollo(T|_R, c|_R)$ does not always exist (see Fig. 2 for an example).

We have shown that given a Dollo-labeling ${\hat{c}}|_R$ for $(T|_R,c|_R)$, we can construct a labeling ${\hat{c}}$ for (*T*, *c*) that yields score $Dollo(T|_R, c|_R)$. It remains to show that ${\hat{c}}$ is a Dollo-labeling for (*T*, *c*). For the sake of contradiction, assume that ${\hat{c}}$ is not a Dollo-labeling for (*T*, *c*). Thus, there exists some other labeling ${\hat{c}}' \ne {\hat{c}}$ that yields score $q < Dollo(T|_R, c|_R)$. In this case, we can use $c'$ to form a labeling for $T|_R$ by propagating the state assignments for vertices of *T* in group 1 back to $T|_R$. This new labeling of $T|_R$ has score at most *q* as follows. Recall that to form $T|_R$ from *T*, we first remove edges associated with maximally-sized subtrees whose leaves are all assigned the ambiguous state; this procedure either has no effect on the score or else decreases it (if any of the removed edges carried substitutions). Second, we suppress vertices (in group 3) with out-degree 1. Consider a pair of vertices *s* and *v* that are incident to the same edge in $T|_R$ but are not incident to the same edge in *T*. If *s* and *v* are in the same state, then the minimum number of substitutions on the path between them in *T* is 0, and if *s* and *v* are in different states, then the minimum number of substitutions on the path between them in *T* is 1. In both cases, the suppression step either has no change on the score or else decreases the score. It follows that ${\hat{c}}|_R$ is not a Dollo-labeling for $Dollo(T|_R, c|_R)$, which is a contradiction.

We now claim that a unique Dollo-labeling ${\hat{c}}_*$ for (*T*, *c*) that can be determined for any vertex $v \in V(T)$ with $SubBip(v) = X|Y$ by applying conditions 1–3 (see proof of Corollary 1) plus one additional condition:

- **Condition 0:** For all leaves $l \in X \cup Y$, $c[l] = \texttt {?}$.

If condition 0 is true, we set ${\hat{c}}_*[v] = \texttt {?}$. If condition 0 is false, we proceed in the usual fashion, setting ${\hat{c}}_*[v] = \texttt {1}$ if at least one of conditions 1–3 is true and setting ${\hat{c}}_*[v] = \texttt {0}$ otherwise. We need to show that this procedure produces a valid Dollo-labeling for (*T*, *c*) for all $v \in V(T)$. This can be achieved by evaluating the outcomes of applying conditions 0–3 to vertices in groups 1–3.

**Group 1:** The outcomes of applying conditions 1–3 to any vertex *v* in group 1 will be the same as applying conditions 1–3 to the corresponding vertex *w* in $V(T|_R)$ because $SubBip(v) = SubBip(w)$ after we remove all leaves assigned state ? from the *SubBip*(*v*).

**Group 2:** Condition 0 will be true for vertex *v* if and only if *v* is in group 2; thus, applying condition 0 assigns state ? correctly.

**Group 3:** Each vertex *v* in group 3 maps to an edge $e = s \mapsto t \in E(T|_R)$. For each of these vertices *v*, the subtree bipartition $SubBip(v) = X|Y$ will have *either* all leaves in *X* assigned state ? *or* all leaves in *Y* assigned state ?. Thus, for any two vertices $v_1$ and $v_2$ in group 3 that map to the same edge $e = s \mapsto t$, $SubBip(v_1) = SubBip(v_2)$ after all leaves assigned $\texttt {?}$ are removed. It follows that $v_1$ and $v_2$ will be assigned the state when applying conditions 1–3. Lastly, we need to show that the state assigned $v_1$ (call *v*) will be the same as either the state assigned to *s* or the state assigned to *t*. For simplicity, assume *v* induces $SubBip(v) = X|Y$ and that all leaves in *X* are assigned state ?; we can now check the outcomes of applying conditions 1–3 to *v*.

- Condition 1 is false because there does not exist a leaf $x \in X$ such that $c[x] =$ 1.
- Condition 2 is false for the same reason.
- Condition 3 may be either true or false.
  - If condition 3 is true for *v*, then *v* is assigned state 1. Because condition 3 is true for *v*, there exists a leaf $y \in Y$ and a leaf $z \in Z$ such that $c[y] = c[z] = \texttt {1}$. Thus, *t* is on the path from the *LCA*(*y*, *z*) to leaf *y*. It follows that *t* is also assigned state 1.
  - If condition 3 is false for *v*, then *v* is assigned state 0. Because condition 3 is false for *v*, at least one of the following statements is true. For all $y \in Y$, $c[y] \ne \texttt {1}$. In this case, *t* is also assigned state $\texttt {0}$. For all $z \in Z$, $c[z] \ne \texttt {1}$. In this case, *s* is also assigned state $\texttt {0}$.
  The above logic can be applied if all leaves in *Y* are assigned state ? (we just swap our arguments for conditions 2 and 3, replacing set *Y* with *X*).

This proves our second claim. Note that our procedure still works when *c* has no missing values because all vertices in *T* will be in group 1. It is also worth noting that the missing states in *c* can be imputed without increasing the Dollo score simply by propagating the states assigned to vertices in group 3 down the tree. In Fig. 2, this would correspond to assigning state 0 to *H*, *I* (because *y* is assigned state 0) and so on. $\square$

The procedure in the proof of Theorem 2 gives an *O*(*nk*) algorithm for determining a unique Dollo-labeling at vertex *v*, denoted ${\hat{c}}[v]$, for a set of *k* characters on *n* species, provided we know *SubBip*(*v*). We refer to this procedure as GetState (see Additional file 1: Algorithm 1 in for details).

The relationship between the subtree bipartitions of a tree and its Dollo-labeling brings us back to the CC-LDP problem, where the solution space is constrained by a set of clades, which in turn constrains the subtree bipartitions of any solution and thus its Dollo-labeling.

### Corollary 2

Consider the set ${\mathcal {T}}$ of all solutions to CC-LDP given species set *S*, character set ${\mathcal {C}}$, and clade set $\Sigma$. Let $\mathcal {STB} = \{ X|Y: X, Y, X \cup Y \in \Sigma \}$ be the set of all subtree bipartitions that can be formed from $\Sigma$, and let $\mathcal {STB}(A) = \{ X|Y \in \mathcal {STB}: X \cup Y = A \}$ be the subset of subtree bipartitions in $\mathcal {STB}$ that are associated with clade *A*. Then, the unique Dollo-labeling at internal vertex *v* in an arbitrary phylogenetic tree $T \in {\mathcal {T}}$ that induces clade *A* must be in the set $Lab(A) = \{ \texttt {GetState}(X|Y, S, {\mathcal {C}}): X|Y \in \mathcal {STB}(A) \}$. Note that if *v* is a leaf, *Lab*(*A*) is simply given by the input character set ${\mathcal {C}}$.

Corollary 2 easily follows from Theorem 2. We refer to *Lab*(*A*) as the *allowed* state assignments for clade *A* and $\mathcal {STB}(A)$ as the *allowed* subtree bipartitions for clade *A*. These quantities can be precomputed or computed on the fly and saved. Whenever this is done, we also compute the set $\mathcal {STB}(A, st)$ of subtree bipartitions *X*|*Y* such that $X \cup Y = A$ and $\texttt {GetState}(X|Y, S, {\mathcal {C}}) = st$.

Lastly, if we know the state assignments for some vertex *v* as well as its children *v*.*left* and *v*.*right*, it is possible for us to compute the number of losses that occur on the outgoing edges of *v*. We simply need to count the number of times *v* is assigned state 1 and *v*.*left* is assigned state 0, repeating for *v*.*right*. This can be done if *O*(*k*) time. We refer to this procedure as CountLosses (see Additional file 1: Algorithm 2 for details). Now we are ready to present the dynamic programming algorithm for CC-LDP.

### DynamicProgramming 1

Let *Dollo*[*A*, *st*] be the smallest number of losses for any pair $(t_A, {\hat{c}}_A)$ such that $t_A$ is a rooted binary tree on leaf set *A* that draws all its clades from $\Sigma$ and ${\hat{c}}_A$ is an assignment of states to all vertices of $t_A$ constrained by Corollary 2 and returning *st* for the root of $t_A$ (note that these requirements imply $A \in \Sigma$ and $st \in Lab(A)$). The quantity *Dollo*[*A*, *st*] can be computed with dynamic programming as follows.

**Base Case:** Clade *A* contains a single species, i.e., $|A| = 1$.

$$\begin{aligned} Dollo[A, st]:= 0 \end{aligned}$$

**Recurrence:** Clade *A* contains multiple species, i.e., $|A| > 1$.

$$\begin{aligned} Dollo[A, st] := \min _{X|Y \in \mathcal {STB}(A, st), St_X \in Lab(X), St_Y \in Lab(Y)}&Dollo[X, St_X] + \\&Dollo[Y, St_Y] + \\&\texttt {CountLosses}(st, St_X, St_Y) \end{aligned}$$

The Dollo score of any solution to CC-LDP equals $\min _{st \in Lab(S)} Dollo[S,st]$, and a solution can be recovered by backtracking.

### Theorem 3

The dynamic programming algorithm above correctly solves CC-LDP.

### Proof

**Base case:** The base case for *Dollo*[*A*, *st*] is trivial because when $|A| = 1$ there is only one rooted, binary tree possible: a single leaf assigned the states given by the input character set ${\mathcal {C}}$. There are no edges and thus no losses, so $Dollo[A, st] = 0$.

**Induction step:** Now we consider the case where $|A| > 1$. By the induction hypothesis, we assume that we have correctly solved $Dollo[X,St_X]$ and $Dollo[Y,St_Y]$. Let $(t_X, {\hat{c}}_X)$ be an arbitrary solution to subproblem $Dollo[X,St_X]$, implying that (1) $t_X$ is a rooted binary tree on leaf set *X* with $Clade(t_X) \subseteq \Sigma$, (2) ${\hat{c}}_X$ is an assignment of states to all vertices of $t_X$ constrained by Corollary 2 and returning $St_X$ for the root of $t_X$, and (3) the number of losses for $(t_X, {\hat{c}}_X)$ equals $Dollo[X,St_X]$, which is the minimum number of losses for any pair (tree and state assignment) that satisfies both (1) and (2). Similarly, let $(t_Y,{\hat{c}}_Y)$ be an arbitrary solution to subproblem $Dollo[Y,St_Y]$. Note that by (1), $X,Y \in \Sigma$ and that by (2), $St_X \in Lab(X)$ and $St_Y \in Lab(Y)$. Let $(t, {\hat{c}})$ be formed by connecting $(t_X,{\hat{c}}_X)$ and $(t_Y,{\hat{c}}_Y)$ at their roots and assigning state *st* to the new root. The pair $(t, {\hat{c}})$ is a candidate solution for subproblem *Dollo*[*A*, *st*] provided that $X|Y \in \mathcal {STB}(A)$ (so requirement 1 is satisfied) and $st = \texttt {State}(X|Y, S, {\mathcal {C}}) \in Lab(A)$ (so requirement 2 is satisfied). These two requirements can be summarized as $X|Y \in \mathcal {STB}(A,st)$. For this candidate solution, the number of losses equals $Dollo[X,St_X] + Dollo[Y,St_Y] + \texttt {CountLosses}(st, St_X, St_Y)$, where the last term gives the number of losses for the new edges. Now we need to consider requirement 3. Specifically, we need to check whether a better score (i.e., lower number of losses) can be obtained from any other candidate solution. Keeping clades *X* and *Y*, other candidate solutions can be formed by all other ways of selecting $St_X \in Lab(X)$ and $St_Y \in Lab(Y)$. Moreover, this process can be repeated for the other allowed subtree bipartitions for clade *A* that produce state assignment *st* (i.e., all other ways of selecting $X|Y \in \mathcal {STB}(A, st)$). Any other possibilities will violate (1) and/or (2), and thus will not produce valid candidate solutions. Thus, our recurrence is correct.

**Recurrence is solvable by dynamic programming:** In our recurrence, *Dollo*[*A*, *st*] only depends on subproblems: $Dollo[X, St_X]$ and $Dollo[Y, St_Y]$ for all $X|Y \in STB(A,st)$, $St_X \in Lab(X)$, and $St_Y \in Lab(Y)$. Since |*X*| and |*Y*| must be less than |*A*|, solving subproblems in order of clade cardinality will guarantee that all trivial subproblems are solved first (hitting the base cases) and that all subproblem dependencies are satisfied moving forward (because there are no dependencies between subproblems corresponding to the same clade but different state assignments at the root). Thus, we can use dynamic programming to solve this recurrence.

**Putting it all together:** We compute *Dollo*[*S*, *st*] for all $st \in Lab(S)$, recording a subproblem for which the number of losses is minimized. Backtracking gives an arbitrary solution to this subproblem, which is also a solution to CC-LDP by (1), (2), and (3). $\square$

### Theorem 4

The runtime of the dynamic programming algorithm described above is polynomial in the number *n* of species, the number *k* of characters, and the number of clades in $\Sigma$. To be specific, it has time complexity: $O(|\Sigma |^{3.726}(n+k) + |\Sigma |^{1.726}nk)$.

### Proof

We need to show that the number of subproblems is polynomial and that each subproblem can be solved in polynomial time (and also that the precomputation phases can be done in polynomial time).

We first consider the number of subproblems. The dynamic programming matrix has two dimensions: the first corresponds to clade $A \in \Sigma$ and the second corresponds to an allowed state assignment $st \in Lab(A)$. The former is clearly $O(|\Sigma |)$. The latter is also $O(|\Sigma |)$ because |*Lab*(*A*)| has an upper bound of $|\mathcal {STB}(A)|$, which in turn has an upper bound of $|\Sigma |$. From this analysis, the number of subproblems is $O(|\Sigma ^2|)$; however, we can tighten this upper bound by using the result from Kane and Tao [35], which gives $\sum _{A \in \Sigma } | \mathcal {STB}(A)| < |\Sigma |^{1.726}$. In other words, the number of subproblems is $O(|\Sigma |^{1.726})$. Note that to perform the traceback, we also need to store pointers back to the two child subproblems for every subproblem but this does not impact the storage or time complexity.

Before tackling the subproblems, we precompute several quantities. First, for each clade $A \in \Sigma$, we compute its associated subtree bipartitions $\mathcal {STB}(A)$; this first precomputation phase can be done in $O(|\Sigma |^2n)$ time using Additional file 1: Algorithm 3 in if performing set operations with bitvectors. Second, for each subtree bipartition $X|Y \in \mathcal {STB}(A)$, we compute its state assignment $st'$ in *O*(*nk*) time using the GetStates function (Additional file 1: Algorithm 1), and then we add $st'$ to set *Lab*(*A*) and add *X*|*Y* to set $\mathcal {STB}(A, st')$, which can be done in *O*(*n*) time and $O(n+k)$ time, respectively, if hashing bitvectors. Therefore, the second precomputation phase can be done in $\sum _{A \in \Sigma } O(|\mathcal {STB}(A)|(nk + 2n + k)) = O(|\Sigma |^{1.726}nk)$ time (see Additional file 1: Algorithm 4 for details).

We now consider the cost of solving each subproblem. Subproblem *Dollo*[*A*, *st*] can be solved by computing candidate solutions from $Dollo[X,St_X]$ and $Dollo[Y,St_Y]$, for all $X|Y \in \mathcal {STB}(A,st)$, $St_X \in Lab(X)$, and $St_Y \in Lab(Y)$. For each candidate solution, we must retrieve $Dollo[X, St_X]$ and $Dollo[Y, St_Y]$, which takes $O(n + k)$ time if hashing bitvectors, execute CountLosses, which takes *O*(*k*) time, and then sum three terms together. Thus, the total time to solve subproblem *Dollo*[*A*, *st*] is

$$\begin{aligned} \sum _{A \in \Sigma }\sum _{st \in Lab(A)}O(|\mathcal {STB}(A, st)||\Sigma |^2(n+k))&\le \sum _{A \in \Sigma } O(|\mathcal {STB}(A)||\Sigma |^2(n+k)) \\&= O(|\Sigma |^{3.726}(n+k)) \end{aligned}$$

because |*Lab*(*X*)| and |*Lab*(*X*)| are both $O(|\Sigma |)$, as previously discussed. Putting this all together, the time complexity of our dynamic programming algorithm is $O(|\Sigma |^{3.726}(n+k) + |\Sigma |^{1.726}nk)$. $\square$

### Extension to Camin-Sokal parsimony

Our results for Dollo can be extended to Camin-Sokal parsimony. Specifically, we can define the *clade-constrained large Camin-Sokal parsimony* (CC-LCSP) problem, in the natural way. We can also extend our algorithm by replacing the CountLosses function to count gains $0 \rightarrow 1$ instead and by redefining the GetStates function based on the following result.

#### Theorem 5

Let *T* be a rooted binary tree, and let *c* be an ordered binary character (with no missing values), both on the same species set. A Camin-Sokal-labeling for (*T*, *c*) must assign state 0 to every internal vertex that is an ancestor of a leaf assigned state 0 and assign state 1 to all remaining vertices.

#### Proof

We first claim that in a valid Camin-Sokal-labeling for (*T*, *c*), any internal vertex that is an ancestor of a leaf assigned state 0 must be assigned state 0. To show our claim, let ${\hat{c}}$ be a Camin-Sokal labeling for (*T*, *c*), let *u* be an internal vertex that is an ancestor of a leaf *l* assigned state 0, and suppose for the sake of contradiction that ${\hat{c}}[u] = 1$. However, since *u* is an ancestor of *l* and $c[l] = 0$, at some point in the path from the *u* to *l* there must be a loss; this is not allowed under Camin-Sokal so we have a contradiction.

We now claim that assigning all remaining internal vertices state 1 will yield the unique Camin-Sokal-labeling for (*T*, *c*). Assume that ${\hat{c}}$ is formed in this way but is not the unique Camin-Sokal labeling for (*T*, *c*). This implies the existence of another labeling ${\hat{c}}' \ne {\hat{c}}$ that yields the same or a lower score for (*T*, *c*). By our first claim, we know that the differences between ${\hat{c}}'$ and ${\hat{c}}$ occur at vertices that are not an ancestor of a leaf assigned state 0, that is, they lie in maximally-size subtrees of *T* whose leaves are all assigned state 1. Thus, in at least one of those subtrees, there exists a vertex *v* such that ${\hat{c}}'[v] = \texttt {0}$, so there are at least two gains in that subtree. Because this subtree is maximally-sized, the parent of its root in the tree *T* must be assigned state 0. Thus, changing the assignments of all vertices in that subtree to 1 will improve the overall score for *T* by at least 1. The same logic can be applied to all subtrees, and re-assigning states in all subtrees from 0 to 1 will yield ${\hat{c}}$. It follows that ${\hat{c}}$ is a Camin-Sokal-labeling for *T* and is the unique Camin-Sokal-labeling for *T*. $\square$

#### Corollary 3

Let *c* be an ordered binary character on species set *S*, let *T* be an arbitrary rooted binary tree on *S*, and let ${\hat{c}}$ be the unique Camin-Sokal-labeling for (*T*, *c*). Then for any internal vertex *v* in *T*, ${\hat{c}}[v]$ can be determined just by having knowledge of *Clade*(*v*); no other knowledge of *T* is needed. Moreover, if *c* is allowed to have missing values, a unique Camin-Sokal-labeling can be found in a similar fashion.

#### Proof

Consider an internal vertex *v* in *T* that induces clade *A*. By Theorem 5, ${\hat{c}}[v] = \texttt {0}$ if the following case holds (otherwise ${\hat{c}}[v] = \texttt {1}$)

- **Case C:** Vertex *v* is an ancestor of at least one leaf assigned state 0.

Looking at clade $Clade(v) = A$, case C holds if condition 4 below is true.

- **Condition 4**: There exists a leaf $a \in A$ such that $c[a] = \texttt {0}$.

To summarize, ${\hat{c}}[v] = \texttt {0}$ if condition 4 is true; otherwise ${\hat{c}}[v] = \texttt {1}$. Condition 4 can be evaluated so long as we know the clade induced by *v* (given the character *c* and its complete leaf set *S*). This proves our first claim.

We now allow for missing values. All aspects of the proof are similar to that for Theorem 2, except we now apply condition 4 instead of conditions 1–3 to vertices in groups 1 and 3 (recall that condition 0 will be true if and only if a vertex is in group 2). Applying condition 4 to vertices in group 1 will simply propagate state assignments from $T|_R$ back to *T*. For vertices in group 3, we need to show that every vertex *v* that maps to the same edge $e = s \mapsto t \in E(T|_R)$ will be assigned the same state: either the state assigned to *s* or the state assigned to *t* (so there may be multiple state assignments that achieve the optimal score). For each of these vertices *v*, the subtree bipartition $SubBip(v) = X|Y$ will have *either* all leaves in *X* assigned state ? *or* all leaves in *Y* assigned state ?. Thus, for any two vertices $v_1$ and $v_2$ in group 3 that map to the same edge $e = s \mapsto t$, $Clade(v_1) = Clade(v_2)$ after all leaves assigned $\texttt {?}$ are removed. It follows that $v_1$ and $v_2$ will be assigned the same state when applying condition 4. Now we just need to check the outcomes of applying condition 4 to $v_1$ (call *v*).

- If condition 4 is true for *v*, it is true for both *s* and *t*. Thus, *v*, *s*, *t* are all assigned state 0.
- If condition 4 is false for *v*, it is false for *t*. Thus, *v* and *t* are both assigned state 1.

This proves our second claim. Note that our procedure still works when *c* has no missing values because all vertices in *T* will be in group 1. $\square$

### Extensions to Fitch parsimony

For the *large* Dollo and Camin-Sokal parsimony problems, the state assignments (labelings) are constrained by the allowed clades (Theorem 2 and Corollary 3); thus we can treat these problems as “stitching” clades together into an optimal scoring tree on the complete leaf set. Extensions of our algorithm to Fitch parsimony are not so straight-forward. Recall that the large Fitch parsimony problem takes unordered binary characters as input and seeks an unrooted binary tree to minimize the Fitch score. To apply our algorithm in this context, it would make more sense to constrain the space with bipartitions, and then to transform the bipartitions into clades by rooting them at an arbitrarily selected leaf; this is the approach taken by ASTRAL [6], which constructs a tree rooted at the arbitrarily selected leaf and then unroots it. Additionally, we would need update the scoring function, computing it as a Hamming distance between state assignments instead of using the CountLosses function for Dollo. The major challenge is how to assign states to the internal vertices for Fitch parsimony. Consider the two trees in Fig. 1E and Fig. 1F, specifically the edge in each tree that induces bipartition *A*, *B*|*C*, *D*, *E*. The Fitch-labeling of the vertex incident to this edge and adjacent to leaves *A* and *B* depends on the remainder of the tree (or at least requires more information about the tree than a single bipartition). Because it is not obvious how to constrain the state assignments at an internal vertex simply by looking at its associated subtree bipartition or clade, we could consider all possible state assignments, which are all binary strings with length *k*. However, in this case, the number of subproblems would become $\Omega (|\Sigma | 2^k)$ rather than $O(|\Sigma |^{1.726})$ for Dollo parsimony, so our dynamic programming algorithm, when extended to the large Fitch parsimony problem, would not run in polynomial time.

## Experimental study

We now describe an experimental study evaluating our dynamic programming algorithm for CC-LDP against traditional methods for parsimony: heuristic search and branch-and-bound.

### Character data sets

We evaluate methods in the context of species tree estimation under the infinite sites plus neutral Wright-Fisher (IS+nWF) model [36, 37]. Under the infinite sites model, characters evolve without homoplasy, meaning parallel mutations and reversals are prohibited. Some types of retroelement insertions, like L1 in mammals [38], are typically assumed to evolve with little homoplasy [39]. The idea is that two insertions are unlikely to occur at exactly the same position in the genome (so no parallel evolution) and that insertions are unlikely to be *precisely excised* (so no reversals) [39]. Note that the absence/presence of an insertion corresponds to ancestral/derived states so these characters are ordered.

Characters that evolve without homoplasy would result in a perfect phylogeny; however, this ignores population-level processes. For sexually reproducing organisms, insertions arising in egg or sperm cells are transmitted from parent to offspring. Thus, the mutation is polymorphic in the population when it arises and its frequency in the population changes randomly assuming neutral evolution (note that the population structure is governed by the species tree). To summarize, an insertion is gained ($0 \rightarrow 1$) exactly once but then it can be lost ($1 \rightarrow 0$) due to genetic drift. These rules are suitable for Dollo parsimony, and indeed, Dollo parsimony has been used to estimate species trees from low-homoplasy retroelement insertions in prior studies (e.g., [18–23]). Here, we re-analyze three retroelement presence/absence data sets; we also benchmark methods on a collection of synthetic data sets simulated under the IS+nWF model.

*Biological Data Sets* The *Myotis* data set from [22] has 11 taxa and 10,595 characters. Each character represents the presence/absence of a Ves SINE (short interspersed nuclear element) insertion at an orthologous position across the species’ genomes. No character states are ambiguous, and all characters are parsimony-informative, specifically there are at least two 0’s and at least two 1’s. The original analysis of this data set included maximum parsimony using branch-and-bound with the Dollo criterion score (Fig. 2 in [22]).

The *Palaeognathe* data set from [17] has 13 taxa and 4,301 parsimony-informative characters (note that 18% of the character states in this matrix are ambiguous). Each character represents the presence/absence of a CR1 LINE (long interspersed nuclear element) insertion at an orthologous position across the species’ genomes. The original analysis of this data set did not include Dollo parsimony; rather insertions were used to corroborate a species tree estimated from (estimated) gene trees with ASTRAL and MP-EST [40].

The *Toothed Whales* data set from [41] has 25 taxa and 1,197 parsimony-informative characters (note that 2% of the states in this data set are ambiguous). Each character represents the presence/absence of a CHR SINE insertion at an orthologous position across the species’ genomes. The original analysis of this data set included maximum parsimony using heuristic search under the Dollo parsimony score; branch support was estimated via bootstrapping with 1000 replicates (Supplementary Figure S1b in [41]).

*Simulated Data Sets* All synthetic data sets used in our study were simulated under an approximation to the IS+nWF model using ms[42]. The simulation requires a model species tree. At the high-level, a gene genealogy is simulated within the model species tree under the coalescent and then a mutation arises on a branch of the genealogy so all taxa that are descendants have the mutation (all other taxa do not). This process is repeated, producing a collection of binary characters that evolved independently within the model species tree. We only utilize the parsimony-informative characters, varying the total number of characters given to methods as input from 500 to 50,000.

The first collection of synthetic data sets are taken from Molloy et al. [43]. These data sets were simulated given the *Palaeognathe* species tree estimated by Cloutier et al. [17] using MP-EST. The ms simulation was repeated 25 times to produce 25 replicate data sets. We created a second collection of synthetic data sets by taking the species trees generated in a prior study [10]. Specifically, Mirarab et al. [10] simulated species trees with varying numbers of taxa under the Yule model with SimPhy [44], setting the species tree height to 2 million generations and the effective population size to 200,000. This process was repeated 50 times for each number of taxa. We ran the ms simulation, described above, for the first 25 species trees with 10, 50, 100, and 200 ingroup taxa (and one outgroup taxa). This produced 25 replicate characters matrices for each number of taxa.

### Methods

We evaluated four different methods for the large Dollo parsimony problem. All approaches implemented in PAUP* [30] were executed using version 4a168_centos64 (downloaded from https://paup.phylosolutions.com).

*Branch-and-bound* Branch-and-bound finds an optimal solution by searching tree space in a systematic fashion. Specifically, the parsimony score of an initial tree is used to rule out parts of tree space that do not need to be searched (as they must contain sub-optimal trees). We used the implementation of branch-and-bound in PAUP* (see Additional file 1: Section 2.1 for command) and saved all optimal trees.

*Fast and Slow Heuristic Searches* FastH is a “fast heuristic search” that operates in two phases. First, a starting tree is constructed via random taxon addition, meaning that the taxa are put in a random order and then a tree is built by iteratively adding these taxa to the tree so that the criterion score is optimized. This process is repeated ten times and then the best scoring tree is taken as the starting tree. Second, hill-climbing is performed from the starting tree with Tree Bisection and Reconnection (TBR) edit moves. FastH was implemented with PAUP* (see Additional file 1: Section 2.2 for the command; the reconnection limit was set to eight branches by default). The 100 best-scoring trees found during the heuristic search were saved for use with our dynamic programming method.

SlowH is a “slow heuristic search” that operates by performing 100 independent searches. In each search, a starting tree is built via random taxon addition and then hill climbing is initiated from the starting tree using TBR edit moves. SlowH was implemented with PAUP* (see Additional file 1: Section 2.3 for the command). As in the FastH the reconnection limit for TBR moves was set to eight branches by default; however, unlike SlowH, FastH only performs a single search, with these edit moves, as oppose to 100 independent searches. All trees with the best criterion score found were saved.

*Dollo-CDP* We implemented our dynamic programming algorithm for CC-LDP in C++ software package Dollo-CDP. The CC-LDP problem requires not only a character matrix but also a set of clades to use as constraints. We evaluated two different approaches for generating the constraints, both of which rely on ASTRAL-III [11]. The idea is to give Dollo-CDP a character set and a set of trees, from which it will generate a set of clusters using ASTRAL-III (note that clusters are subsets of taxa, like clades).

Our two approaches differ in the set of trees given to ASTRAL-III as input. Our first approach (called Dollo-CDP-fast) gives ASTRAL-III the 100 best-scoring trees found by FastH. Our second approach (called Dollo-CDP-char) gives ASTRAL-III the input characters reformatted as unrooted trees, as proposed by [45]. The idea is that each parsimony-informative character encodes an unrooted tree with exactly one internal branch, indicating the transition between taxa in state 0 to taxa in state 1 or vice versa.

After the clusters are computed with ASTRAL-III, Dollo-CDP processes them, keeping only the clusters that form clades in a tree rooted at some set *O* of outgroup taxa (note that outgroups are typically available when using Dollo parsimony, as they are often used when calling variants and coding them as ancestral or derived). A cluster *C* produced by ASTRAL-III is added to the constraint (clade) set if either (1) $C \subseteq O$ or (2) $C \subseteq \{S \setminus O \}$. When the outgroup is a single taxon, both of these approaches ensure a solution to CC-LDP exists. For Dollo-CDP-fast, all trees produced by FastH, denoted ${\mathcal {P}}$, can be rooted at *O*; therefore, $\Sigma = \{ Clade(T): T \in {\mathcal {P}} \}$. For Dollo-CDP-char, a solution is guaranteed by virtue of how ASTRAL-III handles polytomies (i.e., vertices of degree greater than three); however, this produces a large number of clades, which, in turn, makes the second approach more computationally intensive. Consequently, we only apply Dollo-CDP-char to the biological data sets.

The command for running Dollo-CDP is given in Additional file 1: Section 2.4. Users are responsible for providing trees for building constraints if using the first approach. They are also responsible for downloading and extracting ASTRAL-III into the src directory so that Dollo-CDP can find it. We used ASTRAL-III version 5.7.8 from Github (https://github.com/smirarab/ASTRAL).

### Evaluation metrics

All computational experiments were run on the compute cluster for the Center for Bioinformatics and Computational Biology at the University of Maryland, College Park. This is a homogenous compute cluster, with all compute nodes having dual socket AMD EPYC 7313 16-Core processors and two terabytes of memory. All methods were given access to 64 GB of memory, one CPU, and a maximum wallclock time of 24 h (resources were managed by the SLURM submission system). We recorded the total wallclock time in minutes as well as the best Dollo parsimony score found. We added the runtime of FastH to the runtime of Dollo-CDP, when the former was used to construct the constraint set for the latter. Our method Dollo-CDP only counts losses and does not count gains, unlike PAUP*  (note that the number of gains should not impact the relative scores as the evolution of each character must be explained with one gain, either on the tree or implied above the root). To ensure scores were comparable, we recomputed the Dollo criterion score for all trees using PAUP* (see Additional file 1: Section 2.5 for the command).

## Experimental results

We now present the results of our experimental study on biological and synthetic data sets.

### Results on biological data sets

**Fig. 3:**
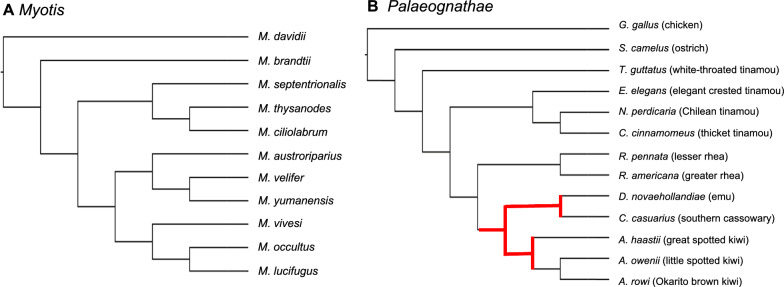
Subfigure **A** shows the tree returned by Dollo-CDP-char for the *Myotis* data set [22]. This is the same tree recovered by branch-and-bound. Subfigure **B** shows the tree returned by Dollo-CDP-char for the *Palaeognathae* data set [17]. A branch-and-bound analysis recovered 60 optimal trees (the Dollo-CDP tree is one of the 60). The three branches highlighted in red indicate the strict consensus of the 60 equally optimal trees

**Fig. 4:**
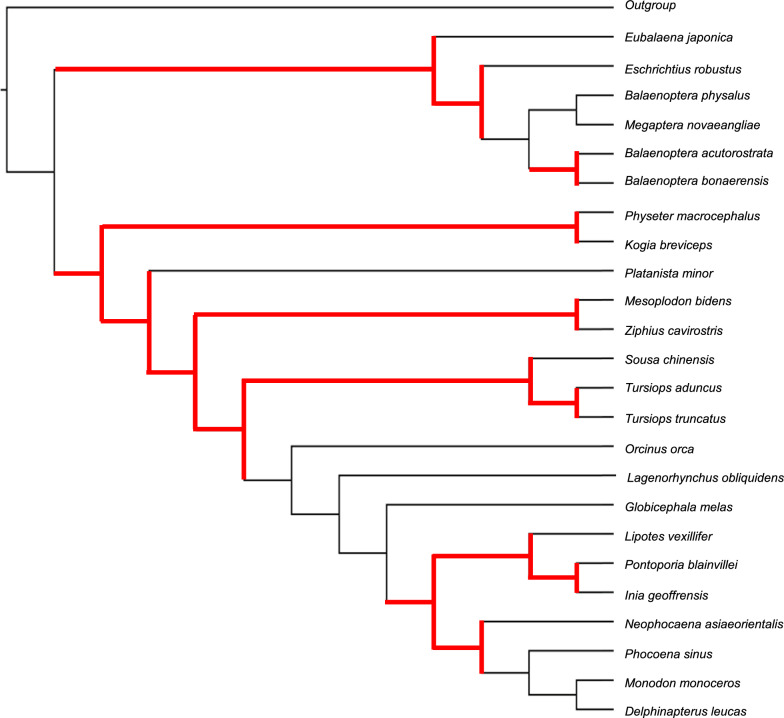
Tree returned by Dollo-CDP-char for the *Toothed Whale* data set [41]. A branch-and-bound analysis recovered 72 optimal trees (the Dollo-CDP tree is one of the 72). The 15 branches highlighted in red indicate the strict consensus of the 72 equally optimal trees

For the *Myotis*, all approaches recovered the same tree as branch-and-bound, which was the unique optimal solution (Fig. 3A). All methods completed in less than a second, except for Dollo-CDP-char (this analysis took 54 s). For the *Palaeognathe* data set, branch-and-bound recovered 60 optimal trees, and the strict consensus had just three branches (Fig. 3B), all of which are in the species tree estimated by Cloutier et al. [17] using MP-EST. All methods we ran recovered one of the 60 optimal trees and completed in less than 3 s. Lastly, for the *Toothed Whales* data set, branch-and-bound recovered 72 optimal trees and the strict consensus had 15 branches (Fig. 4). All methods we ran recovered one of the optimal trees and completed in less than 8 s. To summarize, the biological data sets, which had up to 25 taxa, were small enough to leverage branch-and-bound and the other methods tested achieved similar results to branch-and-bound in terms of parsimony score and runtime.

### Results on simulated data sets

Similar trends to the biological data sets were observed for the first collection of synthetic data sets, which were simulated from a *Palaeognathe* species tree by Molloy et al. [43]. For all but one replicate, all methods recovered trees with the same Dollo parsimony score as branch-and-bound (which typically recovered one or two equally optimal trees). For the remaining replicate, FastH returned a tree with a slightly lower score than the other methods. Thus, Dollo-CDP-fast slightly improved upon FastH in terms of Dollo parsimony score for one replicate. Similar trends were observed for the second collection of simulated data sets with 10 ingroup taxa. Like the biological data sets, the analyses of synthetic data sets suggest that as long as the number of taxa is sufficiently small, all methods will produce similar results and compare favorably to branch-and-bound.

We were unable to run branch-and-bound on data sets with 50 or more taxa (specifically the jobs were killed due to our maximum wallclock time of 24 h). For these data sets, we first looked at whether or not Dollo-CDP-fast found trees with better scores than FastH (Table 1). For 50 ingroup taxa, Dollo-CDP-fast typically improved upon FastH for about half of the replicates. For 100 and 200 ingroup taxa (and at least 5000 characters), Dollo-CDP-fast nearly always improved upon FastH. Moreover, Dollo-CDP-fast has an average runtime of less than 3 min for all data sets (Table 2).

**Table 1:** This table shows a comparison of the tree produced by Dollo-CDP-fast and a best-scoring tree found by the fast heuristic search (FastH)

# of	Same	Dollo-CDP-fast Better
characters	# reps	# reps ($\Delta$ score)
*50 taxa*		
500	16	9 (2)
1000	11	14 (2)
5000	12	13 (7)
10000	14	11 (16)
50000	13	12 (54)
*100 taxa*		
500	25	0 (NA)
1000	22	3 (1)
5000	0	25 (6)
10000	2	23 (13)
50000	4	21 (43)
*200 taxa*		
500	25	0 (NA)
1000	25	0 (NA)
5000	6	19 (2)
10000	4	21 (3)
50000	0	25 (27)

We report the number of replicates for which Dollo-CDP-fast is the same or better than FastH in terms of Dollo criterion score. For the replicates for which Dollo-CDP-fast is better, we also report the average absolute difference in scores between the trees (rounded to the nearest integer). Recall that Dollo-CDP-fast is given trees produced by FastH as constraints; thus Dollo-CDP-fast is strictly slower in runtime and at least as good in score (we confirmed this was the case in our experiments)

Next, we compared the performance of Dollo-CDP-fast to SlowH (Table 2). For 50 and 100 ingroup taxa, Dollo-CDP-fast performed as well as SlowH in terms of parsimony scores and was faster, although SlowH still always completed in less than 16 min on average. For 200 ingroup taxa, SlowH took nearly 40 min on average for 5000-100,000 characters and over 1.5 h for 100,000 characters. In contrast, Dollo-CDP-fast always completed in less than 5 min. On the other hand, SlowH did recover better scoring trees in about one third of the replicates. To summarize, our results suggest that for larger numbers of taxa and larger numbers of characters, Dollo-CDP-fast improves upon FastH and is faster than SlowH.

**Table 2:** This table shows a comparison of the tree produced by Dollo-CDP-fast and a best-scoring tree found by the slow heuristic search (SlowH)

# of	Same	Better	Worse	Dollo-CDP-fast	SlowH
characters	# reps	# reps ($\Delta$ score)	# reps ($\Delta$ score)	Runtime	Runtime
*50 taxa*					
500	25	0 (NA)	0 (NA)	2	8
1000	25	0 (NA)	0 (NA)	2	11
5000	25	0 (NA)	0 (NA)	3	27
10000	24	0 (NA)	1 (14)	4	40
50000	25	0 (NA)	0 (NA)	9	114
*100 taxa*					
500	24	1 (1)	0 (NA)	27	28
1000	24	1 (3)	0 (NA)	23	75
5000	25	0 (NA)	0 (NA)	10	206
10000	24	0 (NA)	1 (7)	13	333
50000	23	0 (NA)	2 (56)	33	935
*200 taxa*					
500	19	5 (1)	1 (1)	131	132
1000	25	0 (NA)	0 (NA)	183	174
5000	17	0 (NA)	8 (3)	77	2358
10000	19	0 (NA)	6 (6)	103	2362
50000	17	0 (NA)	8 (24)	145	5638

We report the number of replicates for which Dollo-CDP-fast is same, better, or worse than SlowH in terms of Dollo criterion score. We also report the absolute difference in scores for the trees estimated by the two methods, averaged over the better and worse cases, respectively (rounded to the nearest integer). Lastly, we report the runtime (in seconds), averaged across all 25 replicates (rounded to nearest integer). Recall that the runtime of Dollo-CDP-fast includes the time to run FastH, the output of which is used to build constraints

## Conclusions

We have introduced the clade-constrained large Dollo parsimony problem and presented a polynomial time algorithm that solves it. Although constraints and dynamic programming (CDP) have been a powerful combination in phylogenetics, to our knowledge this is the first attempt at using CDP for character parsimony. An important distinction between prior problems and character parsimony is the assignment of states at internal vertices required to compute the parsimony score of a tree. We found that Dollo as well as Camin-Sokal criterion scores have nice properties that make CDP possible (they also might make heuristic search quite effective in practice). These nice properties for state assignments did not easily extend to Fitch parsimony, so our algorithmic approach seems less favorable in this setting.

We implemented the CDP algorithm for the Dollo criterion score in a package called Dollo-CDP, including two approaches for generating clade constraints: Dollo-CDP-char and Dollo-CDP-fast. In an experimental evaluation, we found that both approaches had good performance (finding high scoring trees quickly), although all existing methods performed similarly when data sets had relatively few taxa. We found, by way of a simulation study, that Dollo-CDP-fast can provide a benefit for larger numbers of taxa. Most notably, branch-and-bound could not scale to data sets with 50 or more taxa, so Dollo-CDP-fast was the only method run that could provide some guarantee of optimality, albeit a more limited one. In practice, we found Dollo-CDP-fast often found higher scores trees than FastH, even though the trees from FastH  were used to form constraints for Dollo-CDP-fast. SlowH sometimes found higher scoring trees than Dollo-CDP-fast but was much slower for large numbers of taxa and characters. A caveat of our study is that all methods were run with one thread. All searches in SlowH are independent so it would be much faster with threading. Dollo-CDP could also take advantage of threading, using techniques from Yin et al. [46] and could be better optimized. We leave this to future work. Even if the runtime of SlowH improves with threading, Dollo-CDP could then be run using trees found by SlowH, in addition to the ones found by FastH, to form constraints. The benefit here is that leveraging Dollo-CDP is fast, strictly improves upon prior searches, and gives a guarantee of optimality for the constrained solution space.

In analyzing the *Palaeognathae* and the *Toothed Whales* data sets, we found many optimal trees using branch-and-bound. Dollo-CDP returns a single binary tree, and future work should enable users to get a consensus of the optimal trees in the constrained solution space, similar to SIESTA [47]. Lastly, we explored methods for Dollo parsimony in the context of species tree estimation, where data are assumed to follow a population genetics model. Dollo parsimony has also been leveraged in tumor phylogenetics [13–16]. It would be interesting to explore the utility of Dollo-CDP in this application area, especially as the number of leaves (cells instead of species) can be quite large in this setting. Applying Dollo-CDP to this setting will likely necessitate the exploration and development of approaches for generating the constraint set, which impact the performance of Dollo-CDP in terms of score and runtime.

### Supplementary Information

**Additional file 1.** Algorithms and software commands.

## Data Availability

The source code for the DolloCDP software is available on Github (https://github.com/molloy-lab/Dollo-CDP). All analysis scripts and data sets simulated for this study are available on Github (https://github.com/molloy-lab/dollo-study).

## Abbreviation

CC-LDP: Clade-constrained large Dollo parsimony
