# Supplementary material for: Dollo-CDP: a polynomial-time algorithm for the clade-constrained large Dollo parsimony problem
Source: Algorithms Mol Biol. 2024 Jan 8;19:2. doi: 10.1186/s13015-023-00249-9 (PMC10775561; doi:10.1186/s13015-023-00249-9)
Supplement: Supplementary file 1 — Additional file 1. Algorithms and software commands. [file 13015_2023_249_MOESM1_ESM.pdf]

Dollo-CDP: A polynomial-time algorithm for the clade-constrained large Dollo parsimony problem
Supplementary Materials

Junyan Dai, Tobias Rubel, Yunheng Han, and Erin K. Molloy

October 16, 2023

Contents

1 Algorithms 2

2 Software Commands 2

2.1 PAUP* command for running branch-and-bound 3

2.2 PAUP* command for running the fast heuristic search (FastH) 4

2.3 PAUP* command for running the slow heuristic search (SlowH) 4

2.4 Dollo-CDP command 4

2.5 PAUP* command for computing the Dollo criterion score 4

1 Algorithms

Algorithm 1 `GetState`

Input: Subtree bipartition $X|Y$, the set S of n species, and an $n \times k$ character matrix $\mathbf{C}$, with $\mathbf{C}[i, j]$ indicating the state assigned to leaf i for character j

Output: State assignment for subtree bipartition $X|Y$ for each of the k characters in $\mathbf{C}$

function GETSTATE($X|Y, S, \mathbf{C}$)
  $states \leftarrow$ an array of length k containing all `0`'s ; $A \leftarrow X \cup Y$; $Z \leftarrow S \setminus A$
  for $i \in \{0, 1, 2, \ldots, k-1\}$ **do**
    if for all $x \in A$, $\mathbf{C}[x, i] =$ `?` **then** $states[i] \leftarrow$ `?`
    else
      $flag \leftarrow 0$
      if $\exists x \in X$ s.t. $\mathbf{C}[x, i] = 1$ **then** $flag \leftarrow flag + 1$
      if $\exists x \in Y$ s.t. $\mathbf{C}[x, i] = 1$ **then** $flag \leftarrow flag + 1$
      if $\exists x \in Z$ s.t. $\mathbf{C}[x, i] = 1$ **then** $flag \leftarrow flag + 1$
      if $flag \geq 2$ **then** $states[i] \leftarrow$ `1`
  return $states$

Algorithm 2 `CountLosses`

Input: Three k-vectors of state assignments: $statesU$, $statesV$, $statesW$

Output: Number of losses assuming that $statesU$ are associated with a vertex u and $statesV$ and $statesW$ are associated with children of u

function COUNTLOSSES($statesU, statesV, statesW$)
  $nlosses \leftarrow 0$
  for $i \in \{0, 1, 2, \ldots, k-1\}$ **do**
    if $statesU[i] = 1$ and $statesV[i] = 0$ **then** $nlosses \leftarrow nlosses + 1$
    if $statesU[i] = 1$ and $statesW[i] = 0$ **then** $nlosses \leftarrow nlosses + 1$
  return $nlosses$

Algorithm 3 Construct subtree bipartitions from clades

Input: Set Σ of clades

Output: Subtree bipartitions allowed from Σ stored as a dictionary, where $SubBip[A]$ is a list of the allowed subtree bipartitions for clade A (note that only one side of the subtree bipartition is stored)

function CONSTRUCTSUBBIPSFROMCLADES(Σ)
  Sort Σ by cardinality from least to greatest
  for $i \in \{0, 1, \ldots, |\Sigma| - 1\}$ **do**
    $A \leftarrow \Sigma[i]$; $SubBip[A] \leftarrow [\,]$
    for $j \in \{0, 1, \ldots, i-1\}$ **do**
      $X \leftarrow \Sigma[j]$
      if $X \subset A$ **then** $SubBip[A].append(X)$ (already have ptr to $SubBip[A]$)
  return $SubBip$

2 Software Commands

In all commands for heuristic search, we specified seeds so that the results would be reproducible.

Algorithm 4 Dynamic Programming for CC-LDP

Input: Set Σ of clades (sorted by cardinality from least to greatest), a dictionary *SubBip* of allowed subtree bipartitions previously computed from Σ, an $n \times k$ character matrix $\mathbf{C}$, with each character on species set S

Output: Fills in the dynamic programming matrix *Dollo* and the traceback matrix *TraceBack*

```
Lab ← dict()
SubBipByLab ← dict()
for A ∈ Σ do
    if |A| = 1 then
        Do base case for leaves
        st ← C[A, :]
        Dollo[A, st] ← 0
        Lab[A] ← st
        SubBipByLab[A][st] ← ∅
        Traceback[A][st] ← NULL
    else
        Find state assignments for clade A and their associated subtree bipartitions
        Lab[A] ← ∅
        SubBipByLab[A] ← dict()
        for X ∈ SubBip[A] do
            Y ← A \ X
            st ← GetState(X|Y, S, C)
            Add st to set Lab[A]
            Add X to set SubBipByLab[A][st] (initialize if it does not exist)
        For each unique state assignment fill in DP matrix
        bestDollo ← ∞
        bestX, bestY, bestXState, bestYState ← NULL
        for st ∈ Lab[A] do
            for X ∈ SubBipByLab[A][st] do
                Y ← A \ X
                for St_X ∈ Lab[X] do
                    for St_Y ∈ Lab[Y] do
                        cX ← Dollo[X, St_X]
                        cY ← Dollo[Y, St_Y]
                        cA ← CountLosses(st, St_X, St_Y)
                        score ← cX + cY + cA
                        if bestDollo > score then
                            bestDollo ← score
                            bestX ← X
                            bestXState ← St_X
                            bestY ← Y
                            bestYState ← St_Y
            Dollo[A, st] ← bestDollo
            TraceBack(A, st) ← (bestX, bestXState, bestY, bestYState) (two children)
```

2.1 PAUP* command for running branch-and-bound

```
#NEXUS
BEGIN PAUP;
set autoclose=yes warntree=no warnreset=no;
execute <character matrix nexus file>;
outgroup <outgroup name>;
ctype dollo:1-<total number of characters>;
bandb;
```

```
rootTrees;
savetrees File=<output file> root=yes trees=all format=newick;
END;
```

2.2 PAUP* command for running the fast heuristic search (FastH)

```
#NEXUS
BEGIN PAUP;
set autoclose=yes warntree=no warnreset=no;
execute <character matrix nexus file>;
outgroup <outgroup name>;
ctype dollo:1-<total number of characters>;
hsearch start=stepwise addSeq=random swap=None nreps=10 rseed=55555;
hsearch start=1 swap=TBR nbest=100 rseed=12345;
rootTrees;
savetrees File=<output file> root=yes trees=all format=newick;
END;
```

2.3 PAUP* command for running the slow heuristic search (SlowH)

```
#NEXUS
BEGIN PAUP;
set autoclose=yes warntree=no warnreset=no;
execute <character matrix nexus file>;
outgroup <outgroup name>;
ctype dollo:1-<total number of characters>;
hsearch start=stepwise addSeq=random swap=TBR nreps=100 rseed=12345;
rootTrees;
savetrees File=<output file> root=yes trees=all format=newick;
END;
```

2.4 Dollo-CDP command

```
./dollo-cdp \
  -i <character matrix nexus file> \
  -g <outgroup> \
  -t <best 100 trees found from fast heuristic search> \
  -o <output file name>
```

2.5 PAUP* command for computing the Dollo criterion score

```
#NEXUS
BEGIN PAUP;
set autoclose=yes warntree=no warnreset=no;
execute <character matrix nexus file>;
execute <tree nexus file file>;
set criterion=parsimony;
ctype dollo:1-<total number of characters>;
pscores / single=var;
END;
```
